# Experimental Performance Analysis of a Scalable Distributed Hyperledger Fabric for a Large-Scale IoT Testbed

**DOI:** 10.3390/s22134868

**Published:** 2022-06-28

**Authors:** Houshyar Honar Pajooh, Mohammad A. Rashid, Fakhrul Alam, Serge Demidenko

**Affiliations:** 1 Department of Mechanical and Electrical Engineering, Massey University, Auckland 0632, New Zealand; m.a.rashid@massey.ac.nz (M.A.R.); f.alam@massey.ac.nz (F.A.); SDemidenko@Sunway.edu.my (S.D.); 2 School of Science and Technology, Sunway University, Selangor 47500, Malaysia

**Keywords:** blockchain, Hyperledger Fabric, performance, throughput, latency, scalability

## Abstract

Blockchain technology, with its decentralization characteristics, immutability, and traceability, is well-suited for facilitating secure storage, sharing, and management of data in decentralized Internet of Things (IoT) applications. Despite the increasing development of blockchain platforms, there is still no comprehensive approach for adopting blockchain technology in IoT systems. This is due to the blockchain’s limited capability to process substantial transaction requests from a massive number of IoT devices. Hyperledger Fabric (HLF) is a popular open-source permissioned blockchain platform hosted by the Linux Foundation. This article reports a comprehensive empirical study that measures HLF’s performance and identifies potential performance bottlenecks to better meet the requirements of blockchain-based IoT applications. The study considers the implementation of HLF on distributed large-scale IoT systems. First, a model for monitoring the performance of the HLF platform is presented. It addresses the overhead challenges while delivering more details on system performance and better scalability. Then, the proposed framework is implemented to evaluate the impact of varying network workloads on the performance of the blockchain platform in a large-scale distributed environment. In particular, the performance of the HLF is evaluated in terms of throughput, latency, network size, scalability, and the number of peers serviceable by the platform. The obtained experimental results indicate that the proposed framework can provide detailed real-time performance evaluation of blockchain systems for large-scale IoT applications.

## 1. Introduction

Blockchain, originating from Bitcoin [1], encompasses a list of continuously growing data and transaction records, called blocks that are cryptographically linked and secured. Peers maintain the blockchain in a peer-to-peer (P2P) transaction platform, where transactions are recorded in a period of time and packaged into a block by peers to join the blockchain ledger. Blockchain offers a decentralized network with records being tamper-resistant and traceable. Numerous blockchain-based decentralized applications have emerged with the widespread development of this technology. The emergence of Internet of Things (IoT) technology is making large-scale sensor deployment possible at an unprecedented scale for a variety of applications like air quality monitoring [2], healthcare [3], smart homes and smart buildings [4], agriculture [5] and many industrial applications [6]. In all such applications, the typical IoTs are the connected smart devices usually comprising one or more sensors, data gathering and processing controllers with memory, and air or wired interface to the communication channels. As a secure and unalterable architecture, blockchain is a promising paradigm to address the needs of availability, confidentiality, and integrity for IoT applications [7]. The integration of the blockchain to the Internet of Things (IoT) is a challenging enrichment that can guarantee the privacy, security, trust, and data reliability of conventional IoT applications. The feasibility of such blockchain-based IoT systems has been extensively explored recently [8,9]. Nonetheless, the time-consuming consensus process is the primary bottleneck of adopting blockchain technology in IoT applications. The IoT systems are varied in terms of the number of generated requests where applications generate thousands of transactions per second (tps).

Distributed ledger technologies (DLTs) enable the storage of information securely and accurately using a set of cryptographic primitives. Once stored, the information becomes immutable. Hyperledger Fabric (HLF) is a form of a permissioned DLT. It helps enterprises to build their specific DLT solutions more efficiently and securely. The HLF system performance is enhanced by implementing a highly modular framework and pluggable consensus. It can also provide privacy for a broad range of implementation solutions (e.g., IoT networks) while meeting the specific needs of IoT applications. Furthermore, a pluggable consensus approach improves the latency of finality and confirmation. Achieving scalability, throughput, robust cryptographic security, latency, and resource consumption are some of the major challenges while moving from traditional DLT to HLF solutions. The security arrangements of distributed IoT systems can be established and maintained by deploying HLF. HLF offers the implementation of restricted networks and controlled access to user data within the IoT systems.

Permissioned blockchains are those that run a blockchain among a group of known and identifiable members. A permissioned blockchain secures transactions between a set of organizations that have a common aim but do not completely trust each other, such as firms that exchange payments, commodities, or information. A permissioned blockchain can employ classic Byzantine-fault-tolerant (BFT) consensus by relying on the identities of the peers. HLF is the first distributed open-source operating system for deploying permissioned blockchains. Blockchain applications have gained significant attention from both the industry and academia in recent years [10,11]. Such applications are noticeable in different domains ranging from public services [12], finance [13], smart hospitals [14], smart manufacturing [15], supply chains [16], energy trading [17], etc., to the new era of IoT [18]. Despite the development and implementation of many blockchain projects, there are still concerns associated with the blockchain platforms’ throughput, latency, and ability to scale [19].

Performance evaluation presents a significant challenge for current blockchain systems [20,21], particularly during the execution of complex smart contracts. Technical challenges in adopting blockchain systems are associated with parameters such as throughput, latency, scalability, size, bandwidth, security, wasted resources, usability, as well as versioning, and hard forks [22]. Therefore, it is crucial to evaluate the real-time performance of the blockchain platforms. The performance of the blockchain system can be considered overall and as a detailed performance. The overall performance, including the throughput and latency, can help to find the ideal blockchain system that could fit real-world application scenarios. However, the detailed performance computation reveals performance bottlenecks and provides detailed information about the entire process. Blockchain parameters affect the performance, security, and adaptability of the system. This becomes more complicated when choosing an optimal configuration in the IoT systems with vast amounts of small and resource-constraint devices. The parameters need to be validated and tested before deploying the blockchain IoT systems to determine the limitations and possible bottlenecks.

The evaluation of the overall performance of blockchain systems has been studied widely in the literature [23,24,25,26]. However, various process stages still need more detailed performance measurements. There is a lack of metrics to measure and monitor the detailed performance of blockchain systems. Moreover, the scalability and real-time monitoring overhead of the framework need to be comprehensively studied. It is important to be able to monitor the system performance in a large-scale distributed environment. Thus, the way of performance monitoring and the selection of metrics for doing it are the main challenges for the blockchain performance measurements.

HLF network is orchestrated by various components, including endorsers, ordering services, and committers. It constitutes different transaction processing phases consisting of the endorsement, ordering, validation, and commit phases. Therefore, HLF encompasses various configurable parameters such as block size, channels, endorsement policy, and state databases. Finding the right set of values for these parameters is the main challenge in adapting an efficient blockchain system. A comprehensive performance analysis needs to find out an optimal block size to achieve higher throughput and lower latency while considering a more efficient type of endorsement policy in a distributed platform.

This study considers a detailed real-time performance computation model for HLF blockchain systems to address the aforementioned challenges. Albeit there is a possibility to set up the testbed in a physical environment using IoT devices, the reported study set up an HLF multihost network using Amazon Web Services (AWS) because of the flexibility it provides. The testbed was implemented as an “infrastructure” to integrate monitoring tools. AWS supported the deployment of a target network that was a system-under-test (SUT) in the study. It supported a traffic generator such as Tape for HLF to keep sending traffic to the target network/SUT. Flexibility, speed and extendibility to test a large number of nodes were considered with that setup. The private consortium HLF uses Amazon Elastic Compute Cloud (Amazon EC2) to provide a secure and resizable compute environment in the cloud (AWS) for hosting the nodes and simulating the IoT devices which are capable of running the blockchain application [27]. The development of an integrated IoT system using the permissioned blockchain HLF utilising a multilayer blockchain security model to protect IoT networks through a local authentication and authorization mechanism was presented in [8]. The further extension of the research [9] proposed a layered architecture. There, edge IoT devices were implemented in the upper layer along with powerful processors supported by appropriate memory and power resources capable of running HLF for providing security, identity management, and authentication. These edge IoT devises can overcome the limited capabilities of the end IoT devices normally characterized by poor ability to withstand cyber-attacks with their limited CPU processing capability, memory size, and battery power. Raspberry Pi units were employed as end IoT devices capable of running the process and power demanding applications of the Blockchain. Furthermore, the suggested methodology ensured that the data created by the IoT devices were traceable. In addition, the offered method solved the scalability constraints of IoT systems, as well as the processing power and storage concerns of IoT edge devices in the blockchain network. A comprehensive review of blockchain research topics [28] showed that latency, throughput, and scalability were the primary limiting characteristics. The selected performance computation model facilitated collecting real-time performance data by analyzing logs and the daemon process. The study considered the following factors:

1. Distribution. The performance model should consider the real distributed scenario on a large scale.
2. Scalability. The performance monitoring framework should be extended easily to compute the performance of added new peers to the HLF system.
3. Detailed monitoring. The comprehensive analysis of logs could reveal more detailed data associated with various stages of the HLF system.
4. Minimum overhead. The performance computation model should fit real-time monitoring and have a negligible performance impact on the running of the blockchain systems.

The primary focus of this work is to study the impact of the different blockchain network workloads on the performance of the first long-term support release of the Hyperledger Fabric platform v.1.4 [21,29]. For this purpose, an adaptive framework is presented to improve the transaction throughput by redesigning the consensus protocols. At the same time, the expected security and immutability need to be accomplished by the implemented blockchain platform. The aim is to enhance blockchain-based IoT system performance in a distributed environment with a large number of peers. The system performance is evaluated by implementing varying transactions at different rates and block sizes in a large-scale distributed platform. The evaluated performance parameters of the HLF network include latency, throughput, and scalability. In addition, the effect of the endorsement policy is also studied. The following are the main contributions of this paper.

1. Conducting comprehensive experimental analysis of several HLF performance metrics to outline detailed system performance and configuration guidelines to show how various network configurations affect overall system performance.
2. Proposing a scalable model and testing for real-time performance computation of the HLF platform while considering lower overhead and better scalability. The experiments were conducted on networks of different sizes with peers running on multiple hosts to identify the major limitations of the HLF network and performance bottlenecks.
3. Providing comprehensive metrics measurements to analyze and monitor the impact of system parameters (e.g., number of transactions, block sizes, endorsement policies, network sizes) on the HLF performance and scalability in a distributed environment. Highlighting the possibilities and limitations of HLF implementation in large-scale IoT networks.

The experimental study reported in this research performs the scalability analyses for distributed Hyperledger Fabric for a large-scale IoT testbed. Specifically, it performs the scalability analyses for the permissioned HLF blockchain framework for IoT in a distributed computing infrastructure, identifies the bottlenecks to the scalability, and offers possible solutions for addressing them in the context of real-world IoT implementation. Furthermore, this work determines the impact of various blockchain metrics on the system’s latency and resource consumption. This makes it different from the existing studies that do not consider the measurement of the relevant resource utilisation/requirements (e.g., memory and CPU consumption) with increasing numbers of peers in the system. In addition, the interdependency between the scalability of the permissioned HLF blockchain networks and their resource usage was investigated.

The rest of the paper is organized as follows. In Section 2, related works in the area of performance evaluation of HLF blockchain platforms are presented. Section 3 provides an overview of the HLF blockchain technology and the target platforms in this study. Section 4 presents the methodology for evaluating the HLF implementations, key configuration metrics, and experimental setup. A discussion of the obtained results and their implications is covered in Section 5. Finally, Section 6 concludes this paper.

## 2. Related Works

The empirical analysis of DLT has been well documented in the literature evaluating the performance of the implemented blockchain systems such as Hyperledger and Ethereum. Unfortunately, the empirical analysis did not provide standardized results. At the same time, the employed approach showed its flexibility in terms of parameterization. It could find potential bottlenecks and show how to optimize the system performance. A well-controlled test environment can facilitate comparing the performance of different private blockchain platforms and various versions of a particular blockchain system. A number of studies were conducted to evaluate the performance of various hash and encryption algorithms from the data layer in the conceptual model of the blockchain.

The throughput and latency of HLF v1.0 were studied in [30] by deploying an experimental approach using the Caliper [31] benchmarking tool. The study explored the impact of different transactions and various Chaincodes parameters on transaction latency and throughput under microworkloads. The authors evaluated the performance of Hyperledger Fabric characteristics by implementing a varying number of Chaincodes, channels, and peers. The sensitivity of HLF v1.0 throughput to the Orderer setting was highlighted in the evaluation results. Furthermore, the results showed that the HLF v1.0 Committer was incapable of handling the transaction process in parallel using multiple virtual CPUs (vCPUs). That could be considered as a bottleneck in the system performance.

An experimental performance evaluation of two different versions of HLF (v0.6 and v1.0) was conducted by Nasir et al. in [32] to analyze the execution time, throughput, latency, and scalability through implementing various workloads to the system and node scales. The results showed that HLF v1.0 consistently outperformed HLF v0.6 across all measured key performance metrics.

The study [33] evaluated the blockchain latency, throughput, and scalability of the network with varying block sizes, peer CPUs, SSD vs. RAM disk, and various peers. The obtained results identified that the end-to-end throughput of HLF v 1.1 could go up to 3500+ transactions per second (tps) in certain widespread deployment configurations. The latencies were about a few hundred ms while scaling well to 100+ peers.

In [34] the authors conducted a study to find the performance bottlenecks of HLF v1.0 with varying performance metrics consisting of endorsement policies, different block sizes, number of channels, resource allocation, and state database choices (LevelDB vs. CouchDB). The experimental results listed the major system bottlenecks such as endorsement policy verification, sequential policy validation of transactions in a block, and state validation and transactions committing (with CouchDB). The authors outlined optimization solutions to overcome the aforementioned bottlenecks.

The investigation [35] studied the performance of HLF in terms of the underlying communication network using Caliper through the configuration of network parameters, including latency and packet loss in a dedicated testbed. The work examined the impact of transaction rates, network properties, block sizes, Chaincode, and local network impairment influence. The experimental results indicated the transactions’ validation as a significant contributor to the transaction latency in HLF.

In [36], the authors evaluated the performance of the HLF v1.4 platform’ in terms of transaction throughput, latency, and scalability with various network workloads such as a number of transactions, transaction types, and transaction rates.

The impact of malicious behavior on the transaction throughput and latency of HLF performance was explored in the study reported in [37] with designed multiple malicious behavior patterns. The results showed significant degradation in the system performance due to the attack delays and due to keeping some replicas out of working.

Study [38] empirically investigated the performance of the Sawtooth platform. The blockchain platform performance was examined in terms of consistency, stability, and scalability with various workloads and configurations. The authors provided an adjustable optimization approach to configure parameters consisting of the scheduler and maximum batches per block.

Paper [39] proposed a novel design to increase the scalability of blockchain networks. The new design provides satellite chains to construct a network of networks, and it addresses the scalability issue with single Byzantine-fault-tolerant-based (BFT) networks such as HLF v0.6. The proposed architecture allows for safe asset transfers between networks.

Sukhwani et al. [40] provided a performance model for HLF v1.0 based on Stochastic Reward Nets. For each peer in a network, this model can be used to calculate throughput, utilization, and mean queue length. With the help of Hyperledger Caliper, the model was verified. The ordering service and ledger write procedures were determined to be performance bottlenecks by the authors. Larger block sizes, according to the authors, can alleviate the bottleneck at the cost of increased transaction latency. The Hyperledger LTS empirical performance analysis for small and medium businesses are investigated in the study presented by [41].

In [42] a data-sharing system was built using the Hyperledger Fabric. The system performance was evaluated using a variety of sensing data, including images and 3D objects, to reveal the comprehensive performance of blockchain when it is applied to sensor data sharing.

Unfortunately, the reported research did not provide detailed performance computation of real distributed HLF implementation on a large scale and with many peers. For example, the work presented in [43] employed on 8 number of peers (2 peers per organization). Although some of the reported studies tried to configure the HLF platform with a different number of organizations and peers. most considered the performance evaluation within a single host machine and not in a distributed environment. Additionally, the impact of network size on the HLF system overall and detailed performance in different process stages were not investigated comprehensively. Moreover, the aforementioned research methods could not meet the low overhead requirement and have poor scalability. Motivated by this research gap, a comprehensive analysis of the HLF blockchain in a scalable and real distributed environment hosted by various virtual machines was conducted. The main objective was to compare the two different environments (single-host and multiple-host) for implementing HLF. Addressing these challenges, a detailed real-time performance computation and evaluation model for HLF blockchain systems is proposed.

Implementing the permissioned blockchains technology such as HLF on large-scale IoT systems is not a trivial task and is associated with a number of challenges. Unfortunately, it was just partially covered by the existing research studies and technology practices. Aiming to address this deficiency, this work is reporting the results of a comprehensive experimental study focusing on the challenges associated with scalability and performance.

The scalability problem refers to the blockchain system handling an expanding number of peers while continuing to stay operational. The presented results are based on the experimental study involving a large-scale practical setup. They highlight the blockchain network’s scalability issue and demonstrate that it is significantly impacted by the hardware configuration, blockchain system architecture, and complexity of smart contract operations. The performance issue was tackled through the extensive experimental study involving a large-scale distributed computing infrastructure (from 5 to 100 peers) and measuring various performance metrics of a popular permissioned blockchain framework (HLF) under varying conditions.

The throughput, latency, and scalability of a blockchain network depend on the hardware configuration, blockchain network design, smart contact complexity, and operations. The findings reported in this research can serve as a guideline helping to select a suitable hardware configuration, as well as a blockchain network and its parameters supporting a specific blockchain implementations and requirements.

## 3. Hyperledger Fabric

HLF was the first consortium blockchain platform established by the Linux Foundation [43]. HLF supports arbitrary smart contracts (known as Chaincodes [44]) implemented in general-purpose programming languages like Go, Java, and Nodejs. Other existing blockchain platforms deploy smart contracts written in a domain-specific Language (DSL) such as Ethereum’s Solidity to avoid nondeterministic operations. Smart contracts enable a range of HLF applications across various industrial domains. Novel execute-order-validate architecture for transactions and a pluggable consensus protocol differentiates the Hyperledger Fabric from other blockchain platforms. The existing blockchain platforms use conventional order-execute architecture to facilitate the ordering of the transactions based on consensus protocol. After that each peer executes transactions in sequence order. The execution phase increases the network latency. This is because the peers need to scrutinize all transactions in the block and execute them [33]. Key components in the HLF network are peer nodes, clients, and ordering service nodes belonging to different organizations. Each network entity has an identity assigned by a Membership Service Provider (MSP) [45].

**Figure 1:**
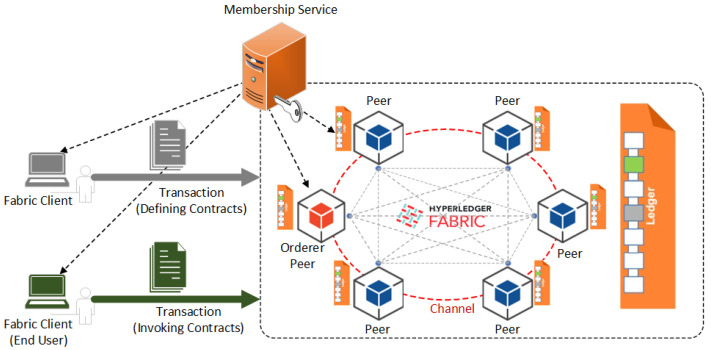
HLF network system architecture with its major components [33].

HLF is a permissioned blockchain platform where the network participants are exposed to each other and fully trusted. Nevertheless, it could be structured based on the governance model constructed so as that trust exists between the participants. Blockchain applications are orchestrated and deployed based on participating organizations within the consortia. Nodes (or peers) host the blockchain and perform smart contract execution as well as mutually maintain the ledger’s state. The concept of the channel in the HLF helps implement the shared Chaincode by all entities or develop private deployment. Chaincodes could be privately shared and deployed to a group of peers making them not accessible by other peers. The data and Chaincode are only available to the participants joining the same channel. The HLF network needs to authenticate and identify the peers through generated cryptographical materials. Therefore, particular channel members can be authenticated in this way. The Ordering Service (OS) performs the ordering of the accepted transactions on a per-channel basis. The overall HLF system architecture is presented in Figure 1.

### 3.1. Transaction Flow in Hyperledger Fabric

**Figure 2:**
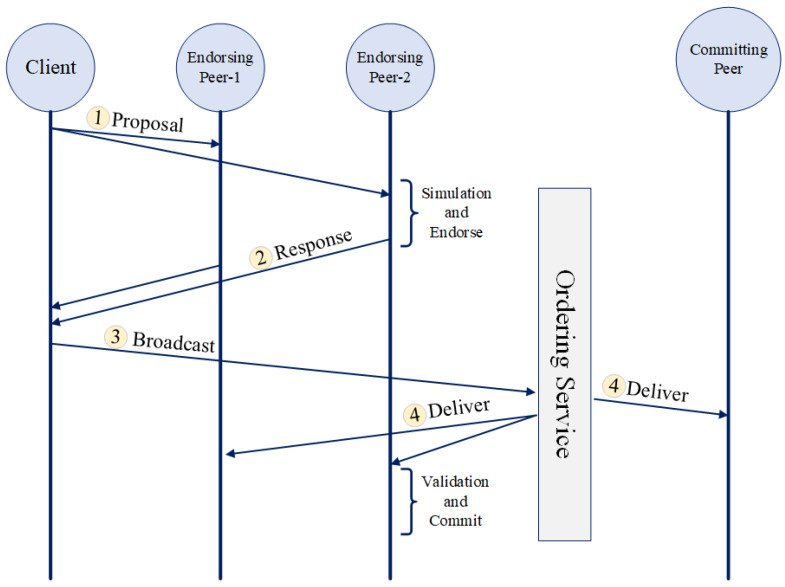
Fabric transaction flow.

HLF employs the execute-order-validate-and-commit transaction model. Figure 2 shows the transaction flow in HFL blockchain platform. It involves three phases: endorsement, ordering, and validation. The transactions are Chaincode invocations running on the Docker [46] container. Thus, it would help to separate them from other running Chaincodes on the same peer and the HLF codes. HLF is a distributed ledger technology where peers keep a copy replica of the ledger. The ledger has two parts: the transaction log and state data. The transaction log keeps track of all the invoked transactions, while the state data represents the current state of the asset at any point in time. Various operations could be carried out on the state data by way of executing the Chaincode. The execution leads to creating transactions in the transaction log. It could also lead to changes in the state data. The transaction log is implemented using the LevelDB, which is a lightweight library for building a key–value data store embedded in HLF peer implementation. The state data consists of the key–value pairs that are versions. The state database is pluggable at the peer level. The LevelDB supports a simple query for key–value pairs. However, it can be replaced with the CouchDB database, a NoSQL database that allows executing complex queries.

Participating organizations, their MSPs, and peers’ identities need to be established before submitting any HLF network transactions. The channel needs to be initialized on the Orderer network with corresponding organization MSPs and organization peers join the channel and initialize the ledger. Finally, the required Chaincodes need to be installed on the channel.

#### 3.1.1. Phase 1: Endorsement Phase

Client applications using the HLF Software Development Kit (SDK) create a request proposal and submit the transactions to endorsing peers based on the endorsement policy embedded in the Chaincode. The client puts a cryptographic signature to the proposal and sends it on the same channel where the Chaincode is deployed. The endorsing peers execute the proposed transactions and corresponding endorsing peers will receive the deterministic output. When installing the Chaincode, an endorsement policy needs to be defined deciding which peers (organizations) have the right to endorse a transaction in the smart contract for it to be considered valid and committed to the ledger. The HLF has multiple steps such as endorsement policies and orderer services to ensure the consensus is met between all the peers. Transactions follow an execute-order-commit flow pattern. Client transactions are first executed in a sandbox to determine their read-write sets, i.e., the set of keys read by and written to by the transaction. Transactions are then ordered by an ordering service and finally validated and committed to the blockchain. This workflow is implemented by nodes that are assigned specific roles. The endorsement response includes the cryptographic materials, response value, and read-write sets generated as a result of Chaincode execution. The endorsing peer signs the transaction response with the peer’s identity through a system Chaincode called ESCC and sends a proposal response to the client. The ledger remains unchanged at this point. The client application collects endorsements from multiple endorsing peers, according to Chaincodes’ defined endorsement policy, and sends them to the ordering service.

#### 3.1.2. Phase 2: Ordering Phase

The ordering service performs transaction verifications. It orders them per channel. The next step is to deliver the ordered transactions by ordering service. All peers need to see a transaction in the same order known as a block formed by ordering service and communicated to all peers. The ordering service deploys one of the developed ordering algorithms, such as Solo, Kafka, or Raft [33]. The Solo ordering algorithm consists of a single ordering service node (that controls all network ordering transactions). It runs on one node and is used for system development. The Kafka is a more distributed algorithm. It deploys the Kafka cluster to create and consume transactions. Thus, it provides Crash Fault Tolerance (CFT) and is recommended in production deployments. The Raft is similar to the Kafka as it follows the leader–follower model. It has the advantage of being CFT. The setup of the Raft is rather straightforward. The ordering service can perform access control permissions of the client nodes to check whether they can broadcast or receive blocks on a particular channel.

The Hyperledger Fabric version 1.4 allows to use SOLO. It is a messaging middleware that is set up with a single node only. No clustering feature is supported in such a setup that is more convenient for a development stage. However, it introduces a single point of failure to the network. In production, developers do not normally consider using SOLO.

The production version of HLF would use Kafka. It is a messaging software that has high throughput and scalability. It is fault-tolerant by a way of clustering. Both Kafka and SOLO support multiple channels making it possible to test out various configurations with multiple channels. A client broadcasts the endorsed transaction to the various peers using the Orderer. The endorsing peer sends back the endorsement responses to the client. The client is ready to send it to the various peers in the network. To be able to perform this action, it invokes the Orderer broadcast service. It sends the transaction in a block to the anchor peers in the member organizations. On receiving the block with the transaction, the anchor peers send it out to the various other peers within that organization. Therefore, the Orderer provides the communication layer for the HLF network. It plays an important role in the consensus process, and it is responsible for managing the order of the transactions.

#### 3.1.3. Phase 3: Validation Phase

Once the block is sent to all peer nodes through either the ordering service provider or gossip protocol (based on gRPC [47] ), the transactions need to be validated. The validation process identifies and rejects invalid transactions. Therefore, only valid transactions are committed and updated in both ledger and world state. The validation phase consists of two consecutive steps: endorsement policy evaluation using validation system Chaincode (VSCC) and read-write conflict check so-called multiversion concurrency control (MVCC). If there is a mismatch in versions and the endorsement policy is not satisfied, the transactions are marked as invalid and their effects are omitted. Valid transactions are the ones that match the read set versions. The peer notifies the client about the success or failure of the transaction. The final step encompasses the ledger update. All the peers commit the identified valid transactions in the HLF network, and its write set is updated in the world state. Thus, in Hyperledger Fabric V1, each transaction goes through three phases: endorsement, ordering, and validation.

## 4. Configuration Parameters and Key Metrics

**Figure 3:**
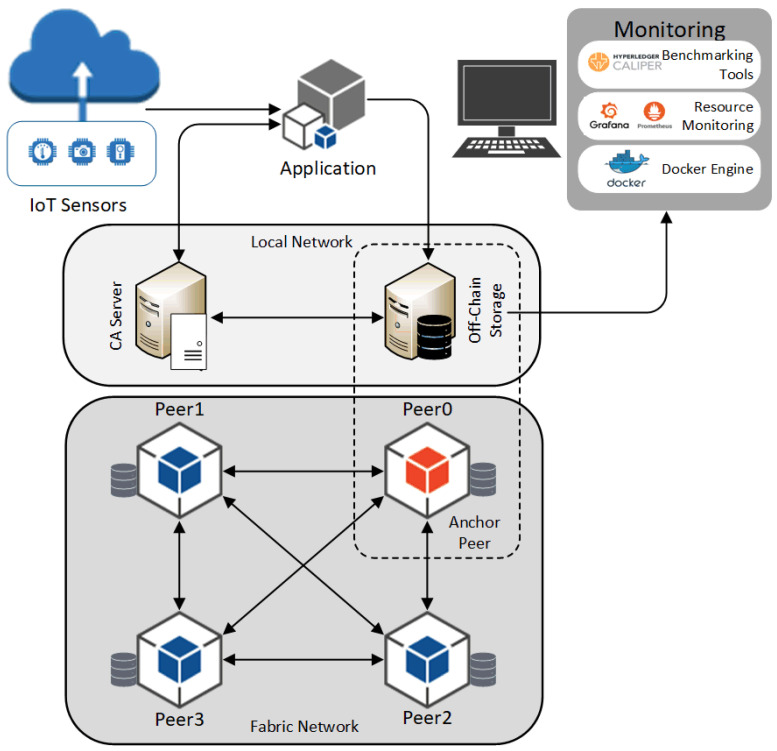
HLF-based distributed system model (the peers and off-chain storage are separated into a scalable platform).

This work aims to study the performance of the scalable HLF in a distributed environment with varying numbers of nodes and various conditions to analyze how specific parameters affect the system performance. The study is limited to a detailed analysis of a few parameters while other aspects are covered in general terms to understand and identify the interplay of network components. Therefore, the primary focus is on studying the overall performance from the peer’s perspective. At the same time, the Orderer and Gossip effects on the experiment were eliminated as they were kept static. The overall system under the test (SUT) and related components are presented in Figure 3. The model includes a single-channel HLF network with one client running benchmarking tools and one anchor peer.

### 4.1. Key Parameters Definition

Several key parameters are considered in this study. The first of them is the block size. An Orderer orchestrates transactions in batches. It then delivers them to peers in a block with the aid of the Gossip protocol. Each peer processes one block of received transactions at a time. The Orderer performs the cryptographical process per block to verify the Orderer signature, while the endorsement signature verification process is handled per transaction. The block size variation influences the throughput and latency. Therefore, this study investigates the effect of various block sizes in conjunction with transaction sending rates. Note that it is assumed here that all transactions are of the same complexity and are independent of each other.

Endorsement policies play a vital role in controlling the number of executions of a transaction and signing the transactions before submission to the Orderer. So, the transaction can successfully be validated by the VSCC phase. The validation confirms that transaction endorsements meet the endorsement policy for that Chaincode (i.e., read/write set does not conflict with simultaneous updates that were committed before.) The time required for the endorsement policy to collect and evaluate transactions is affected by its complexity.

Channel provides an environment where a group of peers creates a separate transaction ledger accessible only by members. However, a peer can join multiple channels and therefore maintain various ledgers. The channels process orders and delivers transactions independently (even though on the same peers). The number of the channels and their functionality directly impact system performance and scalability.

The routine verification process and signature computation by peers as a part of the system Chaincodes need significant CPU and network resources. Running user-defined Chaincodes by endorsing peers during transaction submissions creates extra loads on the system. In the presented case, the design considers a network having low latency and high bandwidth.

### 4.2. Performance Metrics

The Hyperledger Performance and Scalability Working Group developed a document [31] providing precise performance metrics applicable across various DLT platforms. It was used in the reported experiments and analysis.

#### 4.2.1. Transaction Throughput

Deployment, execution, and invoking of smart contracts in different blockchain systems occur at different speeds. It is needed therefore to monitor the transaction throughput. It is measured as the rate of committing valid transactions by the HLF network in a defined period. For the HLF network with a single channel, the measurement at a single peer is considered. However, in the reported model and analysis the experiments were further extended to multiple peers (up to 100). The formal mathematical description of the transaction throughput can be obtained as:

(1) $$TPS_{i}=\frac{Count\;\;(T_{{}_{x}}\;in\;\left(t_{{}_{s}},t_{{}_{e}}\right))}{t_{{}_{e}}-t_{{}_{s}}}$$

where, *T*${}_{x}$ is the total number of submitted transactions, *t*${}_{e}$ is the last block commit time, and *t*${}_{s}$ is the initial transaction submission time. The transaction throughput of *N* peers is calculated by taking the median:

(2) $$\bar{TPS}=\frac{\sum_{i}^{}TPS_{i}}{N}$$

#### 4.2.2. Transaction Latency

When the transaction is sent to the network, it takes some time to be confirmed by the system. Transaction latency is the amount of time taken from the point the transaction is submitted to the point when the transaction is confirmed and committed with the result being available across the network. This metric is measured per transaction. However, in most cases, the experiment provides various statistics on overall transactions such as high, average, low, and standard deviations. In the reported analysis, the transaction confirmations at a single peer and multiple peers with various load levels were checked. The computed end-to-end latency consists of three components: endorsement latency, ordering latency, and commit latency [48]. During a period started at *t*${}_{s}$ and ended at *t*${}_{e}$, the transaction sent to the peer is shown by *Tx*${}_{input}$, and *Tx*${}_{confrimed}$. The average latency of the peer *i* can be computed using the following equation:

(3) $$AL_{i}=\frac{\sum_{T_{x}}^{}\left(t_{Txconfirmed}-t_{Txinput}\right)}{Count\;\;(T_{x}\;\;in\;\;\left(t_{s},t_{e}\right))}$$

The latency of all smart contracts is calculated by taking the median:

(4) $$\bar{AL}=\frac{\sum_{i}^{}AL_{i}}{N}$$

#### 4.2.3. Network Size and Scalability

The implemented HLF network’s ability to support increasing the number of participants is computed in this study. Network size indicates the number of validating peers participating in consensus in the SUT. Network size is presented to show the total number of nodes actively participating in the HLF blockchain network.

#### 4.2.4. Block Size

Block size presents the number of transactions per block, and it is described by three variables: the maximum transaction count, absolute maximum byte, and preferred maximum bytes. The transactions are batched as a block. The study further expands the analysis to include multiple blocks (10 blocks and 50 blocks) in batches. It also studies the effects of different batch sizes on HLF systems.

### 4.3. Test Environment

The primary goal of this study is to benchmark the performance of the distributed HLF implemented on multiple machines. Therefore, an in-depth study of HLF core components and benchmark performance for IoT applications was conducted. The throughput and latency of the SUT were computed by varying configuration parameters listed in Section 4.1 and Section 4.2.

Performance assessment and scalability evaluation of HLF were conducted by deploying different sets of parameters including transaction sending rate, block size, size of the network, and network traffic delivery. To perform the performance evaluation, various metrics have been considered such as network throughput, average transaction latency, and resources. Scalability was measured based on variations in throughput and transaction latency by increasing the size of the network. The test results show the impact of a specific parameter on the performance of the HLF blockchain network, discover the bottlenecks, and illustrate how the adjustments can be deployed to enhance the performance.

**Figure 4:**
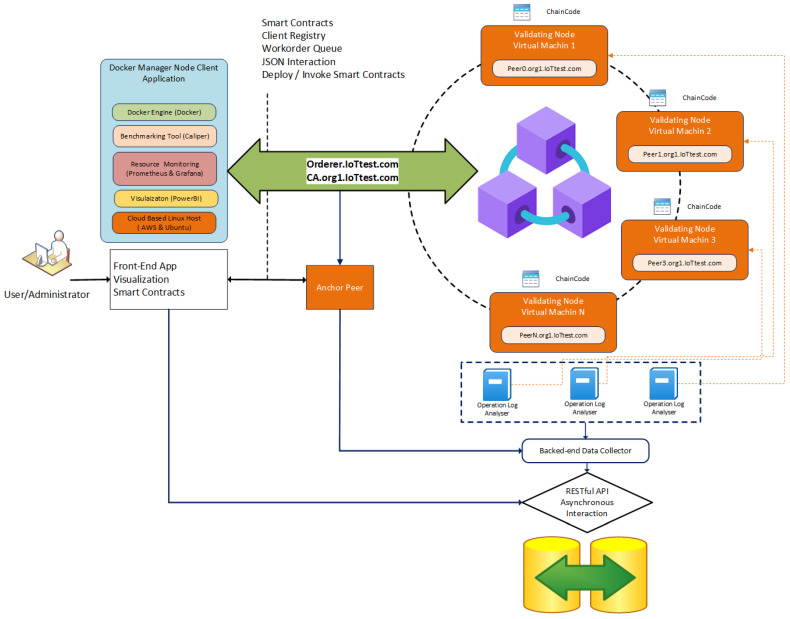
Experimental setup and components for performance evaluation.

Figure 4 depicts the experimental setup model that was used in all experiments. A permissioned HLF network was set up with one organization that includes several peers in each scenario. The ordering service was run on a separate node, and a single channel was implemented. The Chaincode was deployed on the channel to facilitate the assigned tasks.

The setup deployed a private HLF blockchain network in a controlled distributed environment. To achieve realistic results, several Amazon AWS EC2 instances were deployed as an underlying network of nodes. Their parameters are given in Table 1. Each instance was run on its Virtual Machine (VM). All VMs belonged to the same subnetwork to diminish the effect of network latencies within the experiments. The same investigation was conducted several times with different values of peers and nodes. KV denotes a Key Value to be sent as a transaction to the blockchain network.

IoT gateways were modelled as EC2 instances in AWS. Various message transactions were implemented within the IoT system as blockchain transactions. AWS EC2 instance having 2vCPUs, 3.0 GHz Intel Xeon Platinum processors and 4GB RAM was used to run the test benchmark platform. The AWS EC2 instance ran Ubuntu 18.04 LTS and peers, CA, OS, and Caliper with Hyperledger Fabric release v1.4. That test environment was used to investigate the impact of the hardware selection (i.e., CPU and RAM) on the throughput, latency, and scalability of the implemented blockchain network.

Virtual machines as IoT edge (with the exception of the HLF system) aid in reducing traffic load interference generated by numerous systems. The Docker running on VMs (Virtual Machine) are spread over many computers. The number of allowed computers was restricted, even when some Docker systems were installed on a host since a high number of CLIs were necessary for issuing transactions. Each VM had dedicated resources for processing transactions.

The Hyperledger Fabric (version 1.4) framework was deployed to run the blockchain application. It is an open-source permissioned blockchain platform for enterprise applications. Virtual machine instances host Hyperledger Caliper [31], a benchmark tool to measure multiple blockchain performances. The Caliper also runs on client and monitoring instances to broadcast transactions on the HLF channel. The network consisted of numerous peers (from 5 peers per organization and up to a maximum of 100 peers) that were run on scalable network infrastructure. The blockchain components were deployed as a Docker container. Docker Swarm was used to orchestrate and manage the containers spread across the network of VMs. All nodes had the Ubuntu 18.04 LTS operating system.

The Hyperledger Caliper was deployed as a standard open-source benchmarking tool recommended by the Hyperledger community. Further analysis of collected log files and data was conducted using Microsoft PowerBI [49] and Origin Pro [50]. The Prometheus and Grafana [51] were used to monitor Hyperledger Fabric Docker Containers.

The Proof of Work (PoW) consensus shows its robustness. Due to its pseudoanonymous nature, it has been considered the most secure option for cryptocurrency applications. However, in the enterprise ecosystems such as IoT networks and telecom environments, it appeared to be redundant as the blockchain participants are already known to each other. Therefore, permissioned blockchains are designed for enterprise systems that use more straightforward and less resource-consuming consensus protocols, such as Raft [48], that was implemented in this study.

To evaluate the performance of SUT, the following approach is employed. Transactions by the client application are submitted. They change the World State of an HLF network. It leads to testing the performance of consensus. When the application client delivers a transaction proposal to the endorsing peers, this step begins. The endorsement policy determines which endorsing peers are chosen. It is worth noting that the ledger operation (required to activate consensus mechanisms) influences an HLF network’s performance. Therefore, simple transactions have to be implemented to minimize this performance impact. Based on the system time set in Chaincode, each transaction creates a number, which is appended to a value. Each of those values generates a key–value pair including a constant value. The Chaincode is used to write the transactions to the ledger. It runs in a secure and segregated docker container and allows peers to create the transactions. Every transaction is a write transaction that modifies the World State. The endorsers then execute the Chaincode separately, construct a transaction response depending on the execution results, and sign the response. Finally, the application receives the signed transaction proposal response. Because the key’s randomization makes it difficult to write a key that has already existed in the ledger, the transactions are secured against being invalid.

## 5. Results and Discussions

This section presents the impact of various key parameters on the performance of the HLF network. The throughput and transaction latency that are shown here resulted from multiple benchmarking runs while the averages of various runs have also been computed.

### 5.1. Impact of Transaction Sending Rate—Single Host

**Figure 5:**
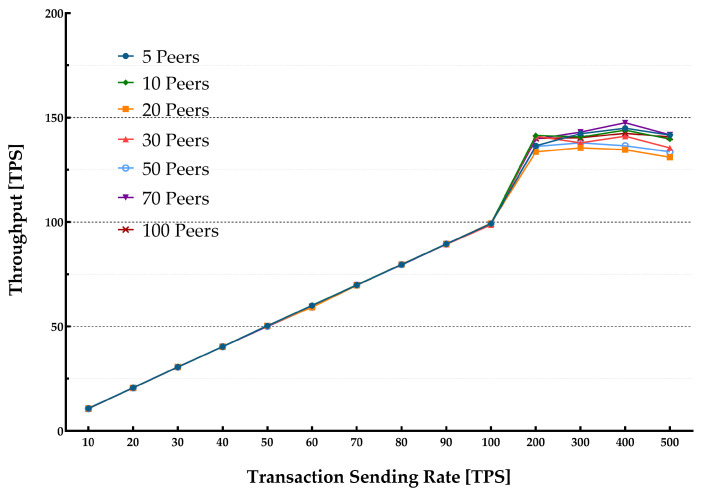
Transactions sending rate vs. throughput—a single host case.

**Figure 6:**
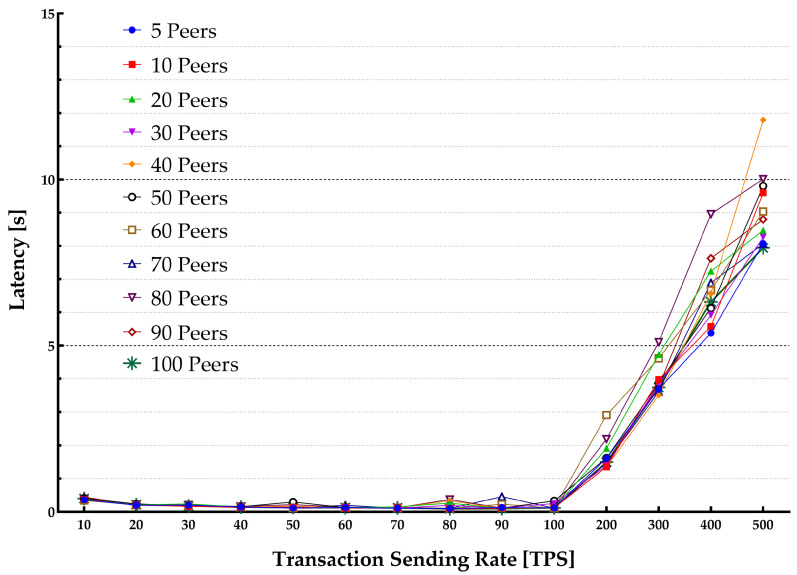
Transactions sending rate vs. latency—a single host case.

Figure 5 plots the average throughput for various block sizes over different transaction sending rates in a single-host setup. Figure 6 presents the average latency over the same transaction sending rates. Table 2 lists the multiple parameters used in the experiment including the transaction sending rate, block size, and a number of peers. Each experiment started with sending transactions with the speed varying from 10 tps up to 500 tps.

The average latency remained below 1s throughout the experiments until it reached around 100 tps. The throughput scaled linearly with the increase in the transaction sending rate. It flattened out at about 100 tps showing the highest usable rate of the system. When the load is increased beyond the peak point, the performance starts to degrade. Additionally, when the number of ordered transactions (waiting in the verification process queue by VSCC during the validation phase) is growing, it significantly increases the commit latency. Therefore, a validation phase can be considered as a bottleneck in the blockchain system thus causing a significant delay. However, the blockchain system depends on SUT hardware capabilities. The growth in the number of involved peers also increases the latency.

HLF relies on the Docker-based architecture. All components of the hyperledger network run in separate containers with no visibility of the neighboring containers. To make them communicate, a network is created, and each container attaches itself to it. This can be found in the docker-compose-cli.yml [46]. In a single host scenario, all containers run on a single machine. The multihost setup includes various node numbers starting from 1 to 100 while the measurements are done in few a steps. By default, the Compose sets up a single network for the app.

During the experiments, an increase in the transaction sending rate was observed. It led to a small growth (about 10%) in the average CPU utilization. The CPU was mainly used during the validation phase of a block by VSCC. It was experimentally found that, for real applications (such as IoT) to achieve lower transaction latency, the use of smaller block sizes with low transaction rates would be preferred. In contrast, higher transaction rates needed larger block sizes to get higher throughput and lower transaction latency.

### 5.2. Impact of Transaction Sending Rate—Multiple Host

Participating entities run peers within the consortium. For larger consortiums, each organization is considered a partner (preferably, they would run at least one peer to contribute to the network). To achieve the real-world production environment, an overlay network of nodes was deployed. Peer nodes were implemented in a distributed system. Using that setup, the effect on system performance of growth of the consortiums’ size was explored. The endorsement policy was configured to help peers endorsing of a transaction on a single channel setup within the consortium and to include the transaction output on the blockchain.

**Figure 7:**
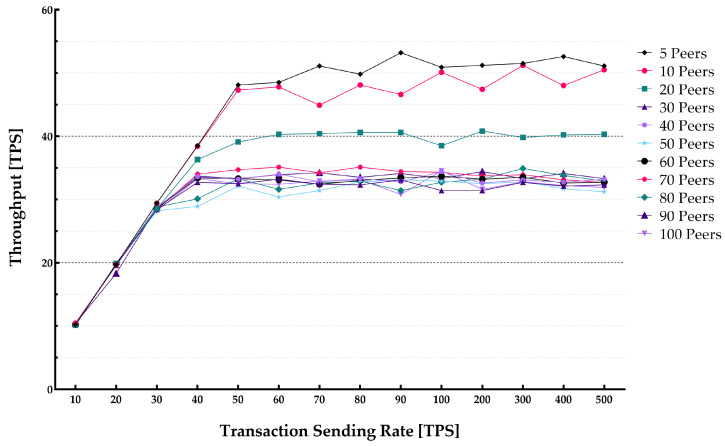
Transactions sending rate vs throughput for multiple host arrangement.

**Figure 8:**
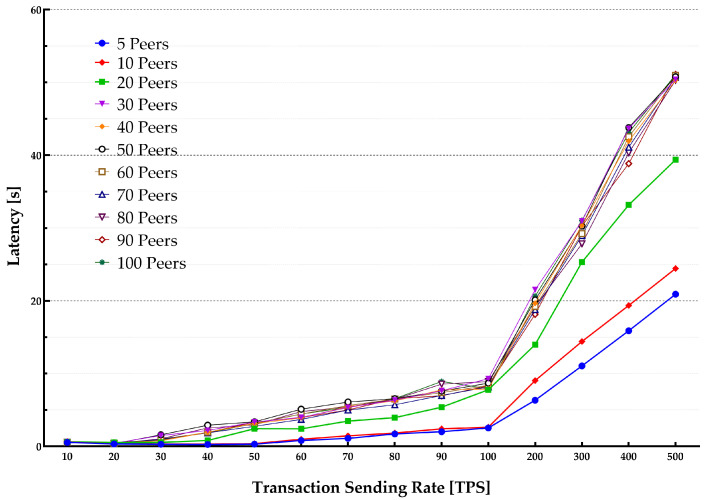
Transactions sending rate vs latency for multiple host arrangement.

Each peer was installed separately in a VM hosted on a commercial cloud provider infrastructure and contributed to the HLF network. The experiment consisted of multiple benchmark rounds with changing transaction sending rates (from 10 tps to 500 tps). Various transactions were generated for each benchmark run and submitted to the HLF network to compute the maximum, average, and minimum of the transaction latency and throughput. Experiments were run with various block sizes in each Section (10 blocks and 50 blocks). Figure 7 and Figure 8 show the throughput and latency measurements for the various workloads.

The observations lead to a conclusion that for a lower transaction rate (100 tps) more involved peers yield a lower throughput and incur higher latencies when the consortium size increases. The results are also slightly different for various block sizes, showing that changing the block from 10 to 50 results in higher latency and lower throughput. The throughput is increased linearly. It could be predicted with an increase in the transaction sending rate until it flattened out at the saturation point, as shown in Figure 7. The results indicate that an increase in the number of peers leads to reaching a lower transaction sending rate peak point. The results for latencies were as predicted.

Figure 8 shows the relation between the transaction latency and the transaction sending rate. The transaction latency is growing linearly with the increase in the transaction sending rate until it reaches its peak. When the system reaches its peak performance, the transaction latency will increase rapidly. At the peak system performance, many transactions will be rejected. At this point, the transaction sending rate surpasses the system capacity of the validate phase. Therefore, the HLF system will reject some transactions. It will also result in increased latency.

### 5.3. Impact of Endorsement Policy

The first experiments considered the Chaincode endorsement policy where peers run on a single host and one peer from the organization endorsed all transactions. Such a representation is for a basic Chaincode implementation between various entities where the organizations have the same authority to control the incoming requests. However, the analysis covered the case when the clients sent the transactions to a number of endorsers with the responses coming from multiple endorsing peers. Hence, the impact of different endorsement policies on the average latency and throughput with varying sending rates was studied. The endorsement policy was configured such that a single peer from an organization provided the transactions endorsement. Then the experiments were expanded by involving more peers endorsing the transactions.

**Figure 9:**
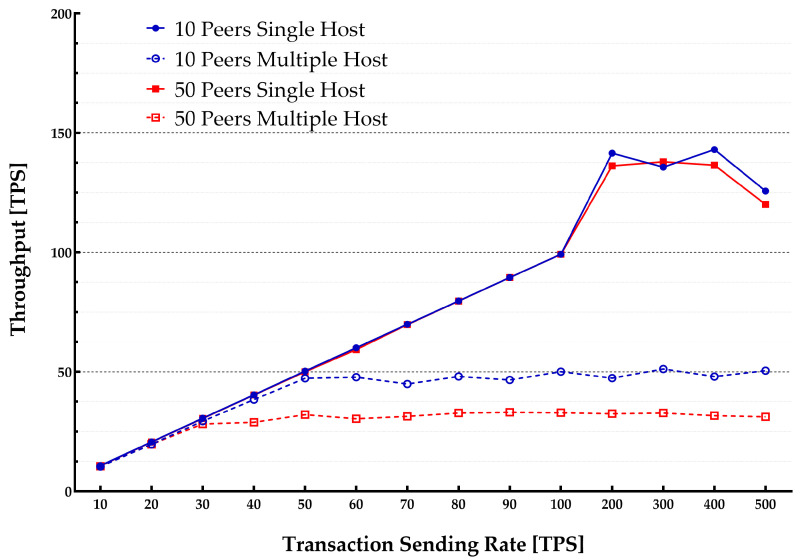
Transactions sending rate vs. throughput for various endorsement policies.

**Figure 10:**
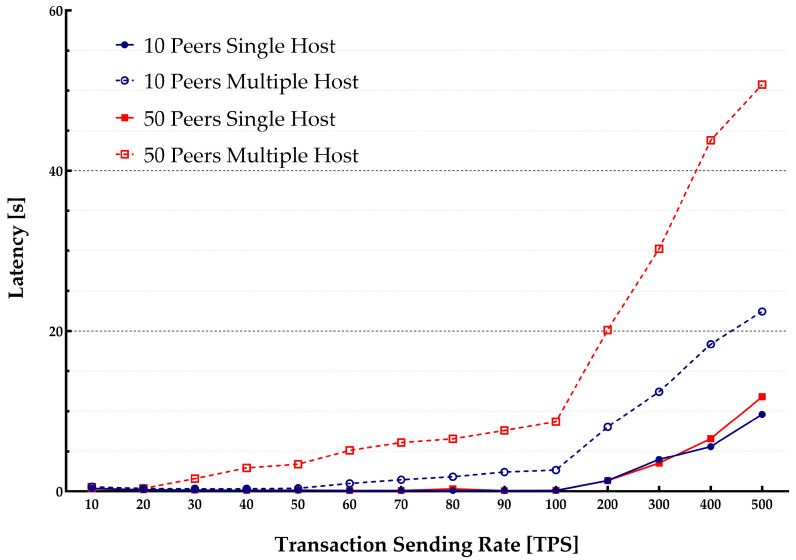
Transactions sending rate vs. latency for various endorsement policies.

Figure 9 and Figure 10 present the resulting throughput and average latency measurements. The results show that both the cases have almost the same latency trend until they reach the peak point. Beyond the peak point, the transaction latency with a single host endorsement indicates a better performance. The throughput increases linearly in all the cases, albeit with different peak points for different configurations. The throughput gets flattened after reaching the peak point, which is not the same for different metric configurations and network setups. The bottleneck can be seen as this version of HLF does not utilize all available CPU cores within participating peers to commit transactions in parallel. Therefore, with higher sending rates, the throughput plateaus to the maximum value that the system can offer.

### 5.4. Impact of Block Size—Multiple Host

A block size dictates the number of transactions batched in the block at the Orderer and delivered to peers through the gossip protocol. The ordering service controls the creation of blocks from the transactions using various parameters such as BatchSize and Batch Timeout. The BatchTimeout indicates the amount of the Orderer waiting time before creating a block regardless of how many transactions are included. The effect of varying block sizes on the throughput and latency with different transaction sending rates was analyzed.

There is a slight increase in the latency for a block size as the transaction sending rate increases closer to the peak point. For smaller block sizes and higher sending rates, blocks are generated faster before the block timeout. Therefore, the transaction waiting time decreases at the ordering service node. The increase in the transaction sending rate means more transactions in a block, and so the time taken by the validation and commit phase is increased accordingly.

**Figure 11:**
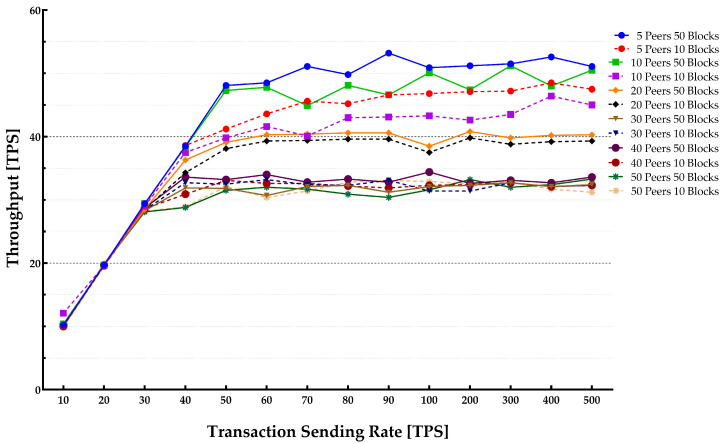
Impact of block sizes on system throughput.

**Figure 12:**
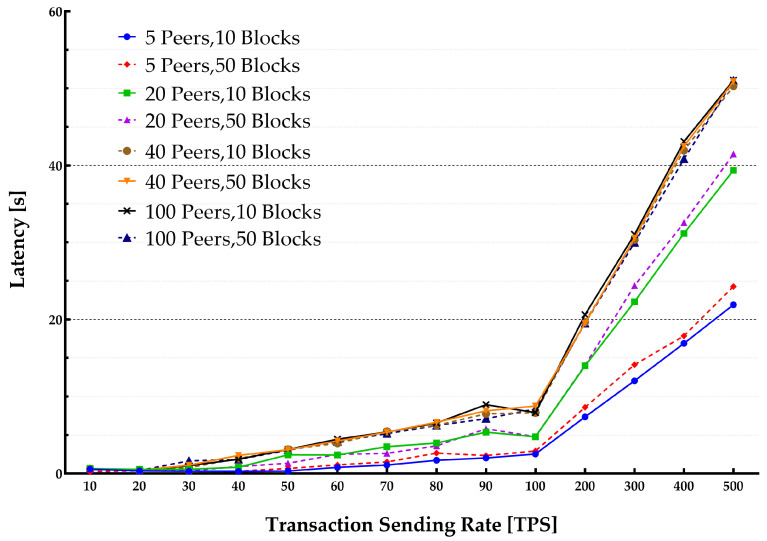
Impact of block sizes on transactions latency.

The results that are shown in Figure 11 and Figure 12 indicate that performance optimization can be achieved with an increase in block size. The block size is indicating the maximum number of transactions in a block that has been published to the blockchain system. The Orderer BatchSize can significantly influence the system throughput. The results show that a smaller block size reduces the throughput. However, these metrics show less deviation with the increase in the number of peers. This metric can be specified during the Orderer bootstrap and can be dynamically altered based on various system applications and the total system load. With the increase in the number of peers, the larger block sizes show less impact on the throughput. Additionally, having transactions with a smaller BatchSize diminishes throughput as a greater number of blocks and block events are required to be generated. These parameters dictate the number and size of transactions in a block.

This fact suggests that when the transaction sending rate is expected to be lower than the peak point, the use of smaller block size is needed to achieve a lower transaction latency for applications. On the other hand, when the transaction sending rate is predicted to be high, the employment of larger block sizes is required to gain a higher throughput and a lower latency.

### 5.5. Impact of a Network Size

This experiment studied the impact of the increase in the network size and the inclusion of more peers. The investigation was done using one organization and one Orderer service node. All transactions were directed to the same Orderer for the validation process. The number of channels remained the same as in the previous experiments, and all Chaincodes on one channel were run. The membership provider was responsible for the permission of entities within the same organization. The main goal was to study the effect of increased peer node number on the total throughput and average latency.

**Figure 13:**
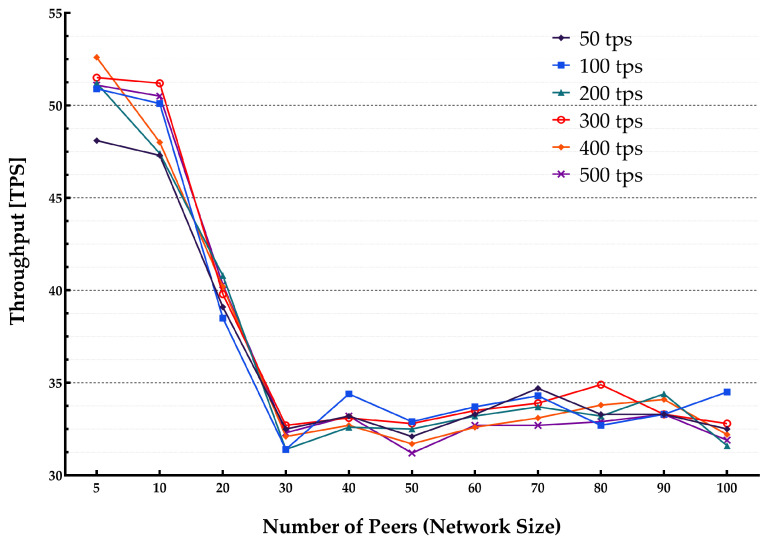
Impact of network size on the system throughput.

**Figure 14:**
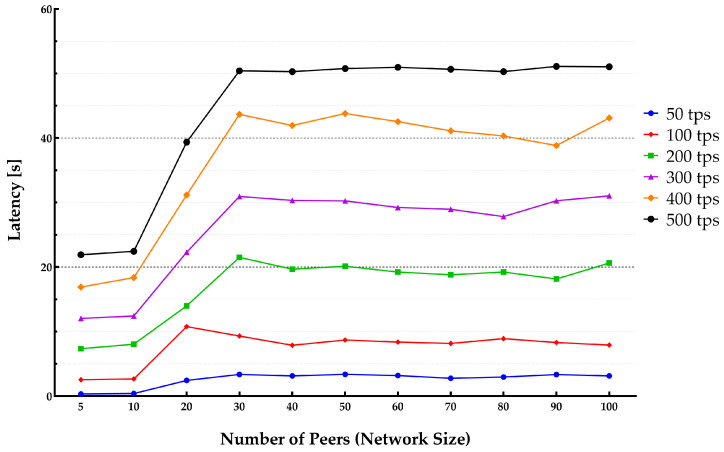
Impact of network size on transaction latency.

The average latency and throughput for network sizes from 5 to 100 peers were studied. The endorsement policy setting consisted of a single channel and single Orderer within the consortium setup to perform endorsement of a transaction to be stored on the blockchain. Figure 13 and Figure 14 show the throughput and average latencies for transactions with varying send rates up to 2500 tps.

It can be observed that for a lower transaction rate below 100 tps, an increase in the number of peers yielded a lower throughput and higher latency when the network size increased. The results slightly varied for different block sizes, showing that changing the block from 10 to 50 results in higher latency and lower throughput.

### 5.6. Resource Consumption

This section presents the results of the study on the consumption of various system resources (CPU, Memory, Disk, and Network Traffic I/O). Such information could be crucial for blockchain users or managers. The major part of CPU resource consumption is accrued during the execution of Chaincodes. It is mainly affected by the business logic implemented in the contract. Complex contracts (including encryption and loops) normally consume more CPU resources. Besides committing the block, the hash of the world state computation also consumes much of the CPU resources.

**Figure 15:**
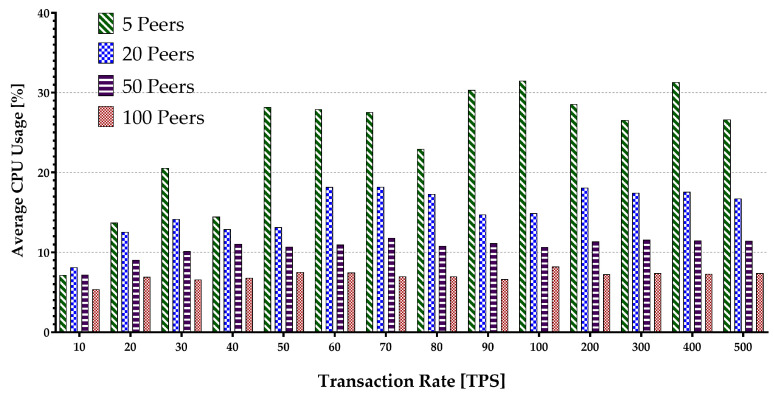
Peers average CPU usage.

**Figure 16:**
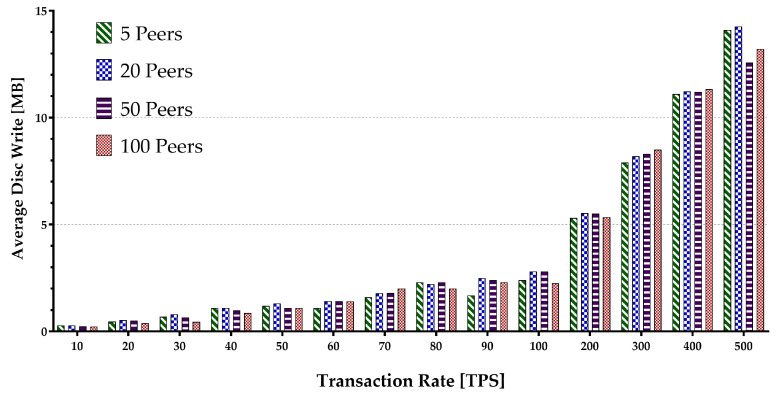
Peers average disk write usage.

**Figure 17:**
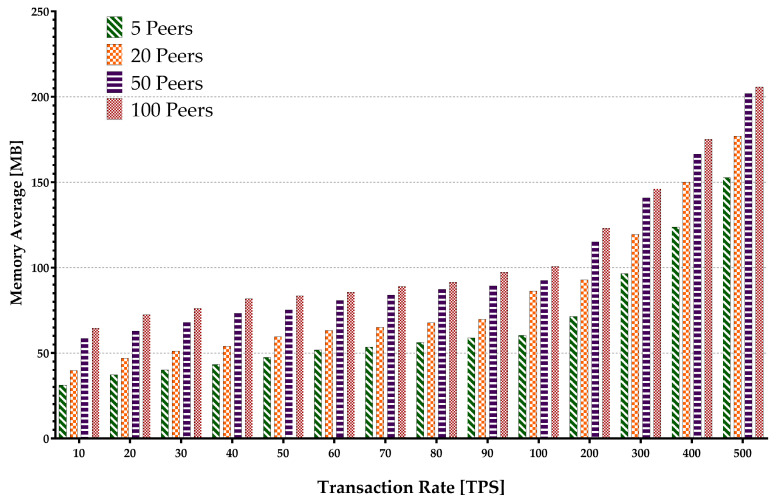
Peers average memory consumption.

**Figure 18:**
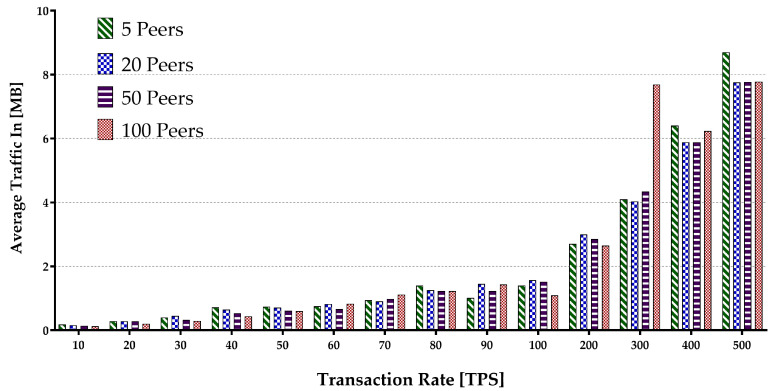
Peers average Network In Traffic.

**Figure 19:**
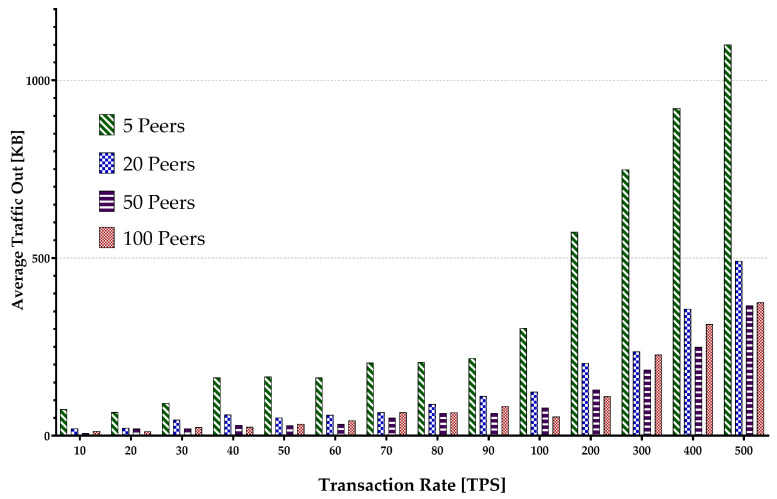
Peers average Network Out Traffic.

Memory consumption occurs when the virtual machine or Docker loads the account data from the world state during the contract execution and opens up some arrays. The hard disk storage space is separated by the blockchain program for storing data, including a world state. Therefore, it uses I/O resources when maintaining the blockchain operations such as block committing and contract execution. Keeping every peer in the same state within different blockchain systems is supported by deploying a different consensus protocol. The consensus protocol performs appending transactions in the network and transfers the block data while consuming the network flow. The experiment encompassed sending multiple transaction batches to calculate the metrics mentioned above. The results are presented in Figure 15, Figure 16, Figure 17, Figure 18 and Figure 19.

Figure 15 depicts the average peer CPU utilization. With the increase in the number of the network peers, more CPUs are involved, and less load is imposed on an individual CPU in general. This results in a decrease in an average CPU utilization. Additionally, the growth in batch sizes increases the CPU usage until it flattens around the peak. The average disk write consumption is shown in Figure 16 indicating the linear growths with both the number of peers and the batch sizes. Figure 17 shows similar patterns of average memory utilization by peers in the network. However, the figure indicates that the maximum batch size suitable for the system is around 100 tps. The data for Network I/O (In/Out) Traffic are presented in Figure 18 and Figure 19, respectively. The average In traffic increases with the increase in the number of peers and batch sizes. However, the Out traffic shows a higher growth with increased batch sizes, with fewer peers involved in the blockchain system.

### 5.7. Summary of Performance Analysis

Numerous scenarios with multiple hosts in the network (from 5 hosts to 100 hosts) were evaluated to analyse the scalability and performance of the Hyperledger Fabric platform in a distributed architecture. The experiments were conducted for the same configuration in a single-host environment. The HLF introduced the multiple organization concept in the Hyperledger Fabric v1.0. The Hyperledger Fabric v1.4 was applied to a multiple-host network of cloud-based instances and analyzed by measuring the system performance metrics such as throughput, latency, network size, resource consumption, and endorsement policies. The results were compared with the single-host deployment. It showed better performance for most of the metrics. However, that scenario was not applicable in production. At the same time, the performance of multiple-host schemes can be increased with multiple-organization deployment where each organization has its dedicated Orderer.

For the single-host, the HLF platform handled a maximum of 25,000 transactions. However, the results showed that the execution time decreased when the number of transactions was more than 100 transactions resulting in a decrease in the throughput and increase in the latency. The single-host implementation had higher throughput and lower latency compared to the multiple-host deployment. For the multiple-host implementation, regardless of the number of peers in the network, the HLF network could handle around 25,000 transactions. However, the number of concurrent transactions was limited and highly dependent on the number of peers in the HLF network. The execution time was higher than in the case of the single-host deployment. This resulted in the Docker deployment in the large-scale network. The throughput was lower, and the latency was higher compared to the single-host option. The Hyperledger Fabric could not instantiate more than 20 endorsing peers on local machines in both scenarios. They needed to be instantiated manually.

The latency grows as the number of nodes and the number of transactions per node increases. This is due to the resource restrictions of the containers that are allocated to the peer nodes in the distributed experiment environment. The minimum latency remains almost constant, as high loads are not imposed on the peer nodes at the beginning. Additionally, the blockchain configuration (e.g., the block size, the number of channels, ordering service, users, endorsing nodes) influences the latency. It can be observed that, in low load cases, all transactions are successfully completed, i.e., no loss of transactions occurs.

According to experimental results, adding peers to the channel reduces throughput and raises average latency. The number of endorsing peers determines the network size. According to the scalability investigation, adding new endorsing peers reduces the blockchain network’s throughput. The rate of executed transactions increases with the increase of the number of peers. The orderers occupy the most significant part of the network resources due to the highly-frequent signaling among the orderers to reach a consensus. Increasing the number of endorsing peers means that each client has to wait for a larger set of endorsements from all the peers to prepare the endorser transaction. Once a block is validated by committing peers, the data records in the state database are updated only with the latest version of data records. Therefore, the data records in the state database are committed, and the number of data records is reduced. The major source of resource consumption in the network is peer and client processes. Overall, the throughput, latency and scalability vary according to the network configuration.

The endorsement process adds significant latency to a transaction. Furthermore, each peer executes the Chaincode in a separate Docker container adding a reasonable performance overhead. In the next step, the client needs to wait for endorsement response from multiple peers to comply with the endorsement policy requirements. Before preparing the endorsement message for the ordering service, the client waits for answers (or timeouts) from all endorsing peers in the current release of Fabric Node SDK. As a result, we cannot use multiple endorsing policies to evaluate the full-system model. This constraint is expected to be addressed with a new feature in the next edition of Hyperledger Fabric. It is worth noting that the transaction latency also depends on the time it takes the Chaincode to execute an endorsement. Therefore, the entire system likely reaches a point where transaction latency becomes untenable because the peers become saturated while consuming all the available CPU and/or disk i/o allocated to the container.

## 6. Conclusions

This paper presented a detailed experimental performance analysis for the scalable Hyperledger Fabric blockchain platform in a distributed large network with varying numbers of nodes and workloads. A scalable framework was proposed for the precise and real-time monitoring of HLF systems. It was characterized by significantly lower overhead compared to previously reported approaches. It also offered more detailed information on various HLF system metrics. Comprehensive performance analysis and evaluation of the well-known HLF blockchain systems was conducted for different network configurations, network load levels, node numbers, and batch sizes. The system performance evaluation was shown in terms of throughput, latency, block size, network size, endorsement policy, CPU usage, memory consumption, disk write, In/Out traffic, and scalability.

The experimental results indicated the feasibility of the proposed framework. At the same time, it has shown that the throughput, latency, and scalability of the blockchain framework depend on hardware configuration, blockchain network design, and smart contract complexity operations. The findings showed that as the number of transactions and batch-timeout rise, latency increases. They also showed that the number of created blocks and the number of transactions per block affected the throughput. Because there are more transactions in a single block, more transactions are validated at the same time resulting, throughput rises as block size grows. Additionally, raising the batch-timeout causes an increase in latency since each block must wait for the timeout even if it has received all of the transactions.

It was found that the throughput of the system linearly increased below the transaction rate of about 100 tps for single-host configuration and around 50 tps for multiple-host arrangement. After the peak point, the throughput saturates and transaction latencies increased showing that the system throughput was sensitive to the Orderer setting. The results also indicate that the change in the number of endorsements influences the performance metrics significantly. The smaller number of endorsements results in better performance. Unfortunately, this also brings security vulnerabilities to the system due to weakening its anticollision properties. One of the main bottlenecks is how the committing peers utilize multiple CPUs present in the system to do parallel transactions that need to be optimized to improve the system performance. The size of transactions significantly impacts throughput and transaction latencies.

Implementing HLF on small IoT devices with limited CPU and power capabilities is generally not too feasible. Currently, available IoT devices are (in most cases) incapable of incorporating blockchain implementation due to their resource limitations. Therefore, a better approach would be to implement the blockchain on edge devices. However, the major bottleneck to the scalability and performance of the HLF networks is associated with the problem of committing peers utilizing present in the system multiple CPUs to do parallel transactions that need to be optimized to improve the system performance. The edge IoT nodes as committing peers need to be equipped with multiple CPUs to mitigate the aforementioned bottleneck. It can be more obvious when the computing workload of the validated IoT edge node is heavy. The experimental results indicate that for incoming blocks (since the committing peer within the distributed environment validates transactions (VSCC) in parallel) there is a significant scalability performance improvement if the committing peer is deployed on a network where peer nodes are occupied with a large number of CPUs. Another significant bottleneck is the endorsement process. Transaction endorsement parallelization can significantly lower the endorser queue length and improve the system’s latency and throughput. Transaction endorsement parallelization can shorten the endorser queue length as well as improve the system’s latency and throughput considerably. One more bottleneck is the transmission of data from the client to the ordering service, as well as ledger writing. The endorsement phase is the limitation of the Hyperledger fabric system when the number of transactions in a block is too large for endorsement. This phase primarily entails the endorsement policy verification and sequence validation of transactions in a block.

Experiments involving more extensive network sizes need to consider the optimized number of endorsements per ChainCode to a smaller subset of peers to achieve better performance. It can be proposed that batch-timeout and block size need to be large for IoT applications with a large number of concurrent transactions in order to maintain high throughput. Batch timeout and block size are to be small for IoT applications with large transactions in order to achieve low latency. Future work will consider implementing the updated version of the HLF and evaluating the use of real-time transactional data, as well as exploring more test cases e.g., analyzing the impact of having multiple Orderers on the overall system performance. Other system configurations such as multiple Hyperledger Fabric organizations and the increase in the number of endorsement peers will be further explored.

## Figures and Tables

**Table 1:** SUT parameters and metrics.

Parameters	Values
Transactions	1 KV write (1-w) of size 20 bytes
Channels	1 Channel
World StateDB	LevelDB
Peer Resources	Up to 100 vCPUs, 3.3 GHz, 10 GiB, Low to Moderate Network Performance
Block Size	30 transactions per block
Batch Timeout	1000 ms
Tx Sending Rate	5–500 (tps)
Number of Blocks	10, 50

**Table 2:** Network nodes and load sizes.

Parameters	Values
Transactions Sending Rate	10, 20, 30, …, 100, …, 500 (tps)
Number of Peers	5, 10, 20, …, 100
Block Size	10, 50

## Abbreviations

The following abbreviations are used in this manuscript:

Chaincode	CC
Crash Fault Tolerance	CFT
Distributed Ledger Technologies	DLTs
Domain-Specific Language	DSL
Gossip Protocol	gRPC
Hyperledger Fabric	HLF
Internet of Things	IoT
Key Value	KV
Membership Service Provider	MSP
Multiversion Concurrency Control	MVCC
Ordering Service	OS
Peer-to-Peer	P2P
Proof of Work	PoW
Software Development Kit	SDK
System Under the Test	SUT
Transactions Per Second	TPS
Virtual Machine	VM
Validation System Chaincode	VSCC
